# When quantum dots meet blue phase liquid crystal elastomers: visualized full-color and mechanically-switchable circularly polarized luminescence

**DOI:** 10.1038/s41377-024-01479-1

**Published:** 2024-06-14

**Authors:** Shan Li, Yuqi Tang, Qingyan Fan, Ziyuan Li, Xinfang Zhang, Jingxia Wang, Jinbao Guo, Quan Li

**Affiliations:** 1 Key Laboratory of Carbon Fibers and Functional Polymers, Ministry of Education, and College of Materials Science and Engineering, Beijing University of Chemical Technology, 100029 Beijing, China; 2 Institute of Advanced Materials and School of Chemistry and Chemical Engineering, Southeast University, Nanjing, 211189 China; 3 Materials Science Graduate Program, Kent State University, Kent, OH 44242 USA; 4 CAS Key Laboratory of Bio-Inspired Materials and Interfacial Sciences, Technical Institute of Physics and Chemistry, Chinese Academy of Sciences, 100190 Beijing, China

**Keywords:** Liquid crystals, Quantum dots

## Abstract

Polymer-based circularly polarized luminescence (CPL) materials with the advantage of diversified structure, easy fabrication, high thermal stability, and tunable properties have garnered considerable attention. However, adequate and precise tuning over CPL in polymer-based materials remains challenging due to the difficulty in regulating chiral structures. Herein, visualized full-color CPL is achieved by doping red, green, and blue quantum dots (QDs) into reconfigurable blue phase liquid crystal elastomers (BPLCEs). In contrast to the CPL signal observed in cholesteric liquid crystal elastomers (CLCEs), the chiral 3D cubic superstructure of BPLCEs induces an opposite CPL signal. Notably, this effect is entirely independent of photonic bandgaps (PBGs) and results in a high *g*_lum_ value, even without matching between PBGs and the emission bands of QDs. Meanwhile, the lattice structure of the BPLCEs can be reversibly switched via mechanical stretching force, inducing on-off switching of the CPL signals, and these variations can be further fixed using dynamic disulfide bonds in the BPLCEs. Moreover, the smart polymer-based CPL systems using the BPLCEs for anti-counterfeiting and information encryption have been demonstrated, suggesting the great potential of the BPLCEs-based CPL active materials.

## Abstract (web summary)

Full-color and “on-off” mechanically-switchable circularly polarized luminescence (CPL) is achieved by incorporating red, green, and blue quantum dots (QDs) into reconfigurable blue phase liquid crystal elastomers (BPLCEs).

## Graphical abstract

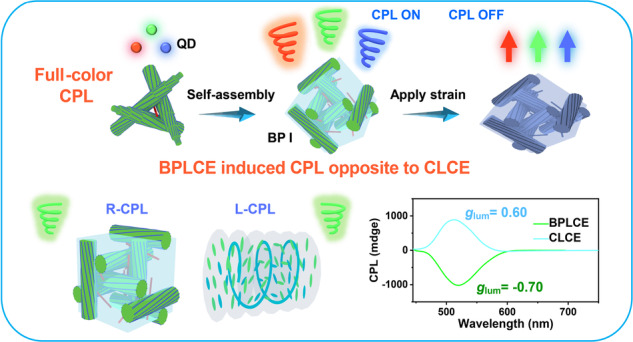

## Introduction

Circularly polarized luminescence (CPL) materials have attracted tremendous attention for their potential applications in many fields^1–5^, such as molecular sensors^6–8^, information encryption^9,10^, and optical storage^11–13^. To quantify the degree of CPL, the luminescence dissymmetry factor is defined as *g*_lum_ = 2(*I*_L_ − *I*_R_)/(*I*_L_ + *I*_R_), where *I*_L_ and *I*_R_ are the luminescence emission intensities of left circularly polarized (LCP) and right circularly polarized (RCP) light. The *g*_lum_ value ranges from +2 to −2, where *g*_lum_ = +2 corresponds to the ideal LCP and *g*_lum_ = −2 is the ideal RCP, respectively. The *g*_lum_ value is a crucial factor for evaluating the performance and potential application of CPL-active materials^14–18^. So far, using cholesteric liquid crystals (CLCs) with helical superstructure has proved to be an effective medium of amplifying *g*_lum_ value^19–22^. The CLCs also exhibit stimulus-responsive properties and can respond to external stimuli, including changes in pH, light, heat, and electric fields^23–30^, which enables the development of responsive CPL materials with potential applications in areas such as anti-counterfeiting and information storage. To date, researchers have incorporated achiral emitters^31,32^, chiral emitters^33–36^, or chiral luminescent LC molecules^37–39^ into small-molecule achiral or chiral LCs to construct CLCs-based CPL-active materials. However, CPL materials constructed by small molecule CLCs are often confined to LC cells, limiting their practical applications in specific scenarios. Compared to small molecular LCs, LC polymers have better processability, thermal stability, and film-forming properties^40–42^, with important applications in anti-counterfeiting and encryption^43–45^. Chiral LC polymers have been viewed as one of the most potential CPL materials^46–49^. Yang et al. incorporated aggregation-induced emission (AIE)-active tetraphenylethylene into CLC polymers and prepared structurally colored polymer films with CPL properties, significantly enhancing fluorescence efficiency^50^. Xiong et al. designed polyether-based CLCs copolymers that demonstrate strong CPL with high intensity by incorporating various dyes^51^. However, these CLCs polymer films have limited responsiveness to external stimuli due to their frozen helical superstructure in solid states^52,53^. Meanwhile, whether in CLC polymers or small-molecule CLCs, magnified *g*_lum_ values of CPL are generally achieved by matching between the emission band and the reflection band of the system; it requires precise modulation of the amount of chiral agent added to the system^54,55^.

Blue phase (BP) is a liquid crystalline mesophase that self-assembles into double-twist cylinder (DTC) intertwined with a 3D lattice of adjacent disclinations between isotropic and chiral nematic phases^56,57^. BP exhibits three subphases, BPIII (amorphous), BPII (simple cubic symmetry), and BPI (body-centered cubic (bcc) symmetry), arranged in decreasing temperature order from the isotropic phase. The structures of BPLCs (BPLCs) exhibit crystal periodicity in the range of several hundred nanometers, allowing them to reflect light within the visible spectrum^58–62^. The unique soft matter properties of BPLCs make them naturally responsive to various external stimuli, such as temperature^63^, electricity^64–66^, and humidity^67–71^. BPLCs have a narrower photonic bandgap (PBGs) and chiral environment, which may be beneficial for improving *g*_lum_ value in the consequent CPL system. Especially, BPLC elastomers (BPLCEs) with elastomeric polymer networks and flexible deformability may be promising candidates for the construction of solid-state CPL-active materials^72,73^.

In this work, we report the fabrication of solid-state CPL-active materials featuring full-color and mechanically-switchable characteristics enabled by novel reconfigurable BPLCEs. Firstly, the CPL signal in BPLCEs is opposite to that in CLC elastomers (CLCEs) despite both having similar CD signals. The strong generation of CPL with high *g*_lum_ values in BPLCEs can be attributed to the unique 3D cubic superstructure and chiral environment, independent of photonic bandgap (PBGs) modulation. Visualized full-color CPL with the largest g_lum_ absolute value of up to 0.74 is achieved by doping red, green, and blue QDs emitters, respectively. Secondly, on-off switching of the CPL signal can be achieved by changing the 3D cubic superstructure of the BPLCEs film through mechanical stretching. Moreover, the programming of CPL signals has been accomplished by utilizing dynamic disulfide bonds in the elastomer network. Finally, we successfully employed QD-BPLCE to achieve visual anti-counterfeiting and information encryption.

## Results

### Fabrication of QD-BPLCE films

**Fig. 1:**
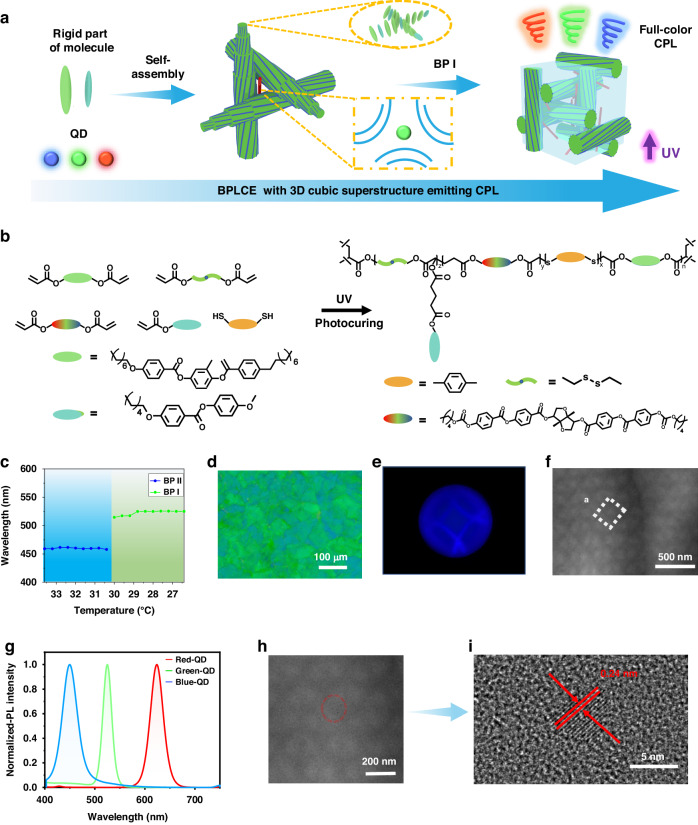
**Fabrication and characterization of QD-BPLCE.** **a** Schematic illustration of QD-BPLCE by molecular self-assembly. **b** Molecular structures used for fabricating QD-BPLCE and schematic process of fabricating QD-BPLCE via one-step in situ photopolymerization reaction. **c** Temperature-dependent Bragg wavelengths for G-G-BPLCE before polymerization. **d** POM image. **e** Kossel diagram and (**f**) TEM image from BPI [110] crystal plane of G-G-BPLCE after polymerization. **g** Fluorescence spectra of QD-BPLCE doped with different QDs. **h** TEM and (**i**) HRTEM images of QDs

The freestanding QD-BPLCE films are prepared by one-step in situ photopolymerization reaction between the acrylate monomers and the dithiol. All the chemical structures of these compounds are shown in Supplementary Information Fig. S1 including LC monomer of RM82 and RM105, chiral dopant of LC756, a thiol crosslinker benzenedimethanethiol (BDMT), a disulfide diacrylate crosslinker disulfanediylbis(ethane-2,1-diyl) diacrylate (DSDA), synthesized from bis(2-hydroxyethyl) disulfide and acryloyl chloride (Supplementary Information Scheme S1 and Fig. S2), photoinitiator of I-651 and oil soluble QDs. The precursor mixtures of QD-BPLCEs were infiltrated into the LC cells and slowly cooled down to form the BPI (for details, see Materials and Methods). The distribution of QDs in BPI is shown in Fig. 1a; QDs accumulate along the disclination lines and interact with LC molecules at the boundaries of disclinations^74,75^. The samples were then exposed to 365 nm UV light for curing at 28.0 °C for 5 min, with an intensity of 30 mW cm^−2^ (Fig. 1b).

As shown in Supplementary Information Table S1, five types of QD-BPLCE with different reflection and fluorescence colors were prepared by adjusting the content of chiral agents and types of QDs. The samples are named based on the reflection color (indicated by the first letter) and the fluorescence color (expressed as the second letter), as follows: R-G-BPLCE, G-G-BPLCE, B-G-BPLCE, G-R-BPLCE, and G-B-BPLCE. During the cooling process, the BPII domains of the precursor of G-G-BPLCE transform into BPI domains (Supplementary Information Fig. S3), resulting in the appearance of reflectance peaks at 456 and 525 nm (Fig. 1c). Figure 1d shows the POM image of G-G-BPLCE, indicating that the self-assembled bcc structure of BPI can be successfully retained after photopolymerization. Figure 1e presents the Kossel diagram of G-G-BPLCE, showing the characteristic diamond-shaped pattern of BPI along the [110] direction. The orientation of the [110] lattice plane of BP I was further confirmed by TEM (Fig. 1f). There was a well-ordered periodic structure with a lattice constant of 265.57 nm (marked) from the TEM image. The QD-BPLCEs were prepared by doped with QDs of different emission wavelengths, which exhibit red (625 nm), green (525 nm), and blue (465 nm) fluorescence, as shown in Fig. 1g. As shown in Supplementary Information Fig. S4, the cross-sectional SEM elemental maps of G-G-BPLCE demonstrate a uniform distribution of QDs. The TEM image in Fig. 1h shows that the QDs are well bound to G-G-BPLCE. The QDs exhibit lattice fringes with a spacing of 0.24 nm according to the results of the high-resolution TEM (HRTEM) image (Fig. 1i). The DSC curve (Supplementary Information Fig. S5) showed that the glass transition temperature (*T*_g_) of the G-G-BPLCE film was about 5.0 °C, indicating their relatively soft behavior at room temperature. In addition, the phase transition temperature from BP to isotropic state (*T*_i_) of the G-G-BPLCE film was about 140 °C. This was further supported by the corresponding POM images (Supplementary Information Fig. S6), which showed that the texture became increasingly blurred and eventually disappeared as the temperature increased from 10 to 140 °C. Figure S7 depicts the reflection and fluorescence spectra of G-G-BPLCE at different temperatures. The sample exhibits good thermal stability, maintaining strong reflectance and fluorescence signals up to 80 °C due to the fully polymerized network.

### CPL emission from QD-BPLCE

The CPL signal of QD-BPLCE films was detected using a CPL-200 spectrometer. We investigated the effect of QD content in G-G-BPLCE, ranging from 0.1 to 1.2 wt%, and plotted the corresponding *g*_lum_ in Supplementary Information Fig. S8. The |*g*_lum_| was found to increase with higher QD concentrations, reaching a peak value of 0.70 at 0.2 wt% QD content. However, excessive QDs impaired alignment and aggregation, leading to a decrease in |*g*_lum_|. Meanwhile, the impact of the G-G-BPLCE film thickness on the CPL signal was also studied (Supplementary Information Fig. S9). With an increase in thickness, the CPL signal initially increased and then decreased, reaching its maximum at 50 μm due to the stronger fluorescence induction when passing through a longer 3D structure. Therefore, the samples containing 0.2 wt% QDs content with 50 μm thickness were selected for investigation for the following experiments. Moreover, possible contributions from linear dichroism caused by macroscopic anisotropy during the CPL measurements were eliminated (Supplementary Information Fig. S10).

**Fig. 2:**
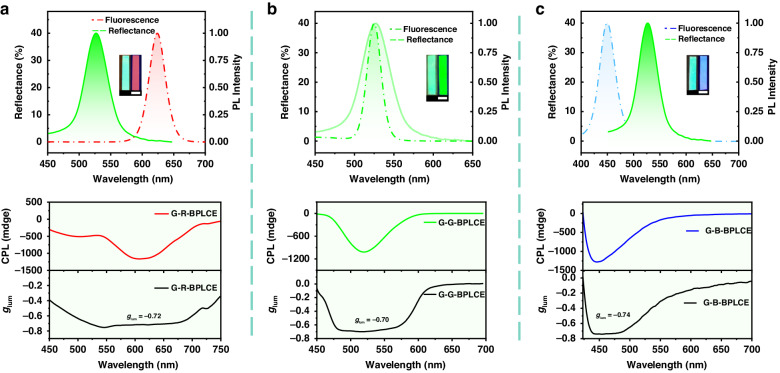
**Full-color CPL emission from QD-BPLCE.** Reflection spectra (solid line) and the PL spectra (dashed line) and CPL spectra and *g*_lum_ curves based on (**a**) G-R-BPLCE, (**b**) G-G-BPLCE, and (**c**) G-B-BPLCE, respectively

The spectra of PL, reflection, and CPL for G-R-BPLCE, G-G-BPLCE, and G-B-BPLCE are displayed in Fig. 2. Full-color CPL was achieved by doping red, green, and blue QDs emitters, and all three samples exhibited strong negative CPL signals. Although the degree of matching between the PBGs and fluorescence emission varies among the three samples, their maximum |*g*_lum_| values are all similar, around 0.7. This indicates that the polarization degree of CPL signals in BPLCE is unrelated to the location of PBGs.

**Fig. 3:**
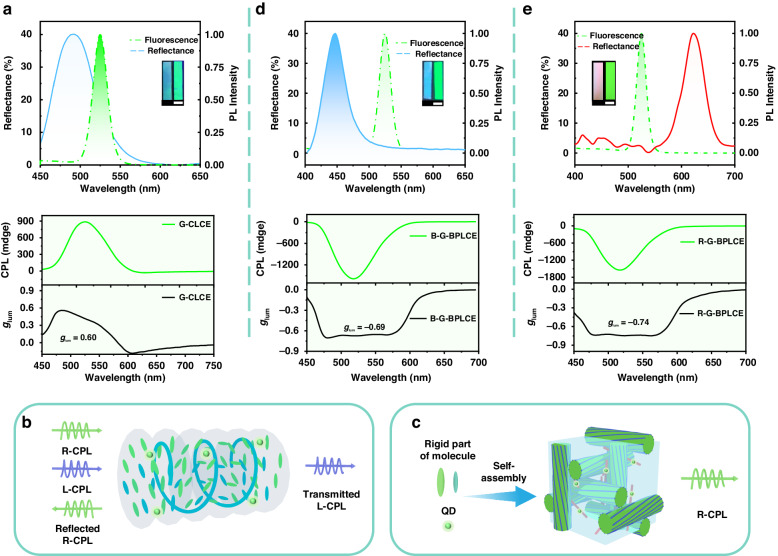
**CPL emission from QD-BPLCE and CLCE.** **a** Reflection spectrum (solid line) and the PL spectra (dashed line) and CPL spectra and *g*_lum_ curves based on CLCE. Schematic diagrams of CPL signal generation by (**b**) CLCEs and (**c**) BPLCEs. Reflection spectra (solid line) and the PL spectra (dashed line) and CPL spectra and *g*_lum_ curves based on (**d**) B-G-BPLCE and (**e**) R-G-BPLCE. Inset: photograph the samples taken under UV light (right) and natural light (left). Scale bar = 1.0 cm

To compare the differences in CPL signal generation between BPLCEs and CLCEs, a G-CLCE film was prepared by polymerizing the same proportion of G-G-BPLCE precursor mixture at the cholesteric phase temperature of 22 °C. As shown in Fig. 3a, G-CLCE exhibits a solid positive CPL signal with a maximum *g*_lum_ of 0.60. The CD signals of G-G-BPLCE and CLCE are depicted in Fig. S11. Due to the use of the chiral dopant LC756, the samples exhibit strong negative CD signals. The peaks of the CD signal correspond to the PBG. The CD signals of G-G-BPLCE and CLCE are similar, while their CPL signals are opposite, demonstrating that the mechanisms inducing CPL signals in BPLCEs and CLCEs are not the same. Figure 3b illustrates the principle of generating CPL signals from CLCEs. In particular, right-handed CLCEs selectively reflect RCP and transmit LCP. When the PBGs of the CLCEs partially or completely match the emission spectrum of the luminescent molecules, the excited right-handed CPL is reflected. At the same time, only the generated left-handed CPL is transmitted. Consequently, right-handed CLCEs generated a left-handed CPL signal. However, selective reflection is not the cause for inducing CPL signals in BPLCEs. As well known, BPLCEs exhibit a highly ordered 3D structure and a strongly chiral environment. Once the QDs are in the BPLCE mixture, they participate in the self-assembly process with the molecules to form supramolecular 3D structures. As a result, right-handed BPLCEs induce a right-handed CPL signal, as depicted in Fig. 3c.

To verify this deduction, further analysis was conducted to investigate the impact of different PBGs of BPLCEs on the CPL signal. Figure 3d, e shows that both R-G-BPLCE and B-G-BPLCE exhibited negative CPL signals. Despite changing the PBGs of QD-BPLCE, the corresponding CPL signal peaks remained relatively stable when the same amount of QDs was incorporated. These observations suggest that the CPL signal detected in QD-BPLCE is induced by the chiral structure of BPLCEs, resulting in the detection of a right-handed CPL signal independent of any changes in the PBGs of BPLCEs.

### Mechanically tuning CPL emission in QD-BPLCE

Next, we investigated the mechanical response of CPL emission from QD-BPLCE. Taking the R-G-BPLCE as an example, the film exhibits excellent stretching capabilities, with a maximum deformation capacity of up to 110% (Fig. S12). The measured modulus for the R-G-BPLCE was 3.7 MPa. The reflection spectrum of R-G-BPLCE continuously shifts from red (625 nm) to blue (465 nm) (Δ*λ* ≈ 160 nm) as the applied strain increases from 0 to 100% (Fig. S13). Stretching-releasing cycles were performed over 10 times (Fig. S14), and no noticeable degradation was observed.

**Fig. 4:**
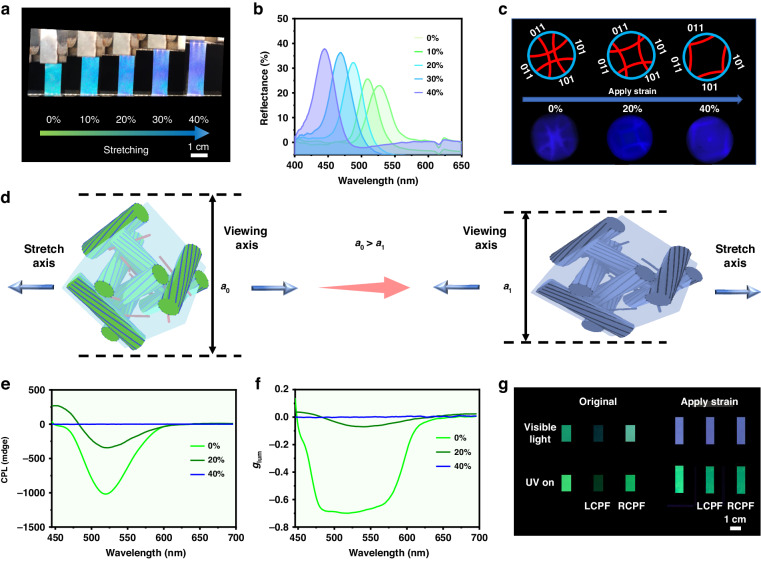
**Mechanically tuning CPL emission in QD-BPLCE.** **a** Photograph of a G-G-BPLCE film being mechanically stretched. **b** Reflection spectra of the G-G-BPLCE film at various strains (from 0 to 40%). **c** Kossel diagrams and the corresponding schematics of the G-G-BPLCE film at different strains (from 0 to 40%). **d** Schematic diagrams of a typical deformation of the BPI unit cell. CPL spectra (**e**) and *g*_lum_ curves (**f**) of G-G-BPLCE at various strains (from 0 to 40%). **g** Photographs of a G-G-BPLCE film at 0 and 40% strain under visible light and 365 nm irradiation with no polarizer, RCPF, and LCPF

When using G-G-BPLCE film, its color changes continuously from green to blue upon mechanical stretching (Fig. 4a) at 25 °C. Figure 4b shows the reflection spectra of the G-G-BPLCE film at various strains, and it can be observed that the reflectance peak wavelength continuously shifts from 525 to 468 nm simultaneously (Δ*λ* ≈ 57 nm). Figure 4c illustrates the changes in the Kossel patterns during the stretching process. The [110] crystal plane of the BPI is observable before stretching. The application of lateral force compresses the transverse plane, causing the Kossel lines corresponding to the [101] and [011] planes to slowly move out of the field of view when the applied strain increases from 0 to 40%. The observed mechano-chromic behavior is attributed to the lateral stretching force, which reduces the thickness of the layer. Consequently, there is a decrease in the effective periodicity of the photonic crystal lattice along the viewing direction due to the approximate volume conservation of the material (Fig. 4d). Figure 4e, f shows the CPL signal and *g*_lum_ of G-G-BPLCE at different degrees of deformation, respectively. At 20% deformation, the maximum |*g*_lum_| is only 0.0645, approaching 0 at 40% deformation. These results show that external mechanical stimulation can lead to the disappearance of the CPL signal in QD-BPLCE. Stretch-induced disappearance of CPL cycles was performed over 10 times (Fig. S15), and no noticeable degradation was observed. We propose that the destruction of the 3D chiral structure of the QD-BPLCE during the stretching process is the underlying cause of the disappearance of the CPL signal. The reflectance spectra change in G-G-BPLCE during the stretching process under right-handed circularly polarized filter (RCPF) and left-handed circularly polarized filter (LCPF) are shown in Supplementary Information Fig. S16. As the strain increases, there is a rapid increase in left circular polarized reflectance, which is analogous in many respects to the 1D case of CLCEs. The disappearance of the CPL signals due to mechanical force is also visually detectable. Due to the robust CPL signal of QD-BPLCE, the entire process is visualized. In its initial state, without CPF or with RCPF, G-G-BPLCE exhibited strong green CPL (Fig. 4g). With LCPF, the polarization states of the CPL signal can be easily distinguished. Under a 40% strain, no significant difference is observed under RCPF and LCPF, indicating the disappearance of CPL in G-G-BPLCE.

## CPL regulation by dynamic disulfide bond in QD-BPLCE

**Fig. 5:**
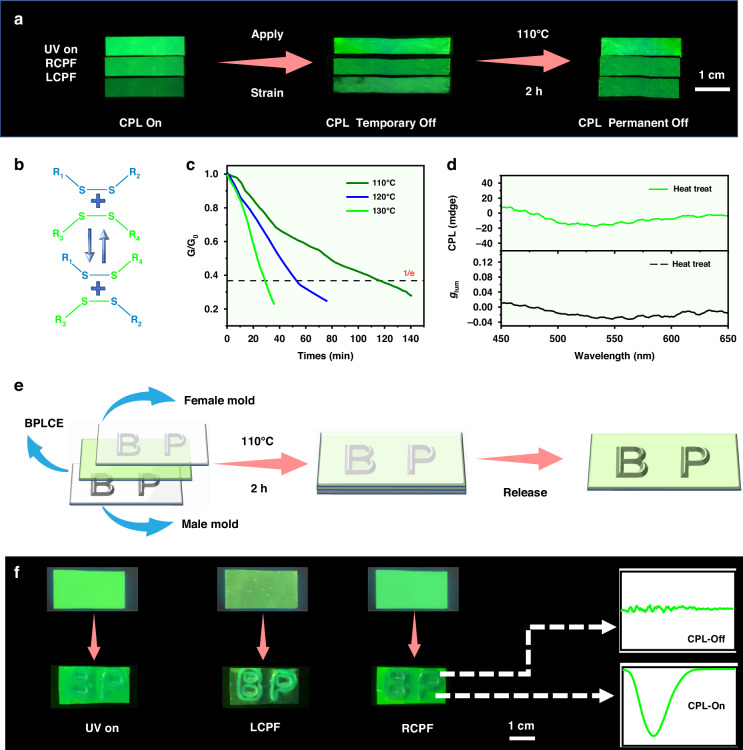
**CPL emission in QD-BPLCE after heat treatment.** **a** Photograph of a G-G-BPLCE film by applying a strain of 40% followed by disulfide bond-exchange reaction at 110 °C. **b** Schematic diagram of G-G-BPLCE based on disulfide bond-exchange reactions. **c** Normalized stress relaxation for G-G-BPLCE at various temperatures. **d** CPL spectra and *g*_lum_ curves of G-G-BPLCE after heat treatment. **e** Schematic illustration of writing information onto a G-G-BPLCE film using male and female molds. **f** Photographs of a G-G-BPLCE film before and after information writing under 365 nm irradiation with no polarizer, RCPF, and LCPF

The disappearance of the CPL signal induced by mechanical force is temporary. When the external force is removed, QD-BPLCE automatically returns to its initial state, and the CPL signal reappears. In our work, by activating the dynamic disulfide bonds within the QD-BPLCE, lattice changes induced by stretching can be fixed, ultimately leading to the permanent extinction of CPL signals. As shown in Fig. 5a, G-G-BPLCE film was uniaxially stretched by about 40% at room temperature and then kept at the fixed length for 2 h under a temperature of 110 °C Due to thermally activated exchange reactions of disulfide bonds, the polymer network can adapt to the new arrangement. After cooling G-G-BPLCE back to room temperature and removing it from the stretching frame, the similar emission intensity observed in the RCPF and LCPF of G-G-BPLCE indicates the disappearance of the CPL signal. As depicted by the Raman spectrum of QD-BPLCE in Fig. S17, it reveals a characteristic vibration peak at approximately 507 nm, which corresponds to the typical position of a disulfide bond, indicating the successful introduction of disulfide-bonds into QD-BPLCE. Figure 5b illustrates the rearrangement mechanism based on dynamic disulfide bonds in a QD-BPLCE. The network structure of QD-BPLCE utilizes dynamic disulfide bonds, which gives QD-BPLCE topological structure rearrangement ability at high temperatures. Stress relaxation experiments on QD-BPLCE films were carried out at varying temperatures (110, 120, and 130 °C) to explore the exchangeable reaction of disulfide bonds. As shown in Fig. 5c, the stress of the film rapidly relaxed at high temperatures, and the stress relaxation speeded up as the temperature increased. As shown in Fig. S18, the relaxation time τ* was used to calculate the activation energy (*E*_a_ ≈ 85.2 kJ mol^−1^) according to the Arrhenius relationship. Moreover, a small quantity of catalyst facilitates the welding process between samples (Figs. S19 and S20). After heat treatment, the CPL signal in G-G-BPLCE almost disappears, and the |*g*_lum_| is nearly 0 (Fig. 5d). Thus, activating dynamic disulfide bonds successfully obtained QD-BPLCE films without CPL signals. Furthermore, information writing in QD-BPLCE was achieved using an on/off system with a positive/negative mold designed to inscribe the letters “BP”, as shown in Fig. 5e. When G-G-BPLCE is inserted into the mold, the compression of its longitudinal lattice eliminates its CPL signal and forms the letters “BP”. Applying pressure and heating at 110 °C for 2 h leads to a dynamic exchange of disulfide bonds, which fixes the temporary shape. After cooling to room temperature and opening the mold Under RCPF, a weak CPL pattern of the letter “BP” can be observed (Fig. 5f). This means the CPL signal disappears only in the stretched part, while the CPL signals in the remaining portions remain unchanged (Fig. S21).

## Multiple-mode QD-BPLCE for anti-counterfeiting and information encryption

**Fig. 6:**
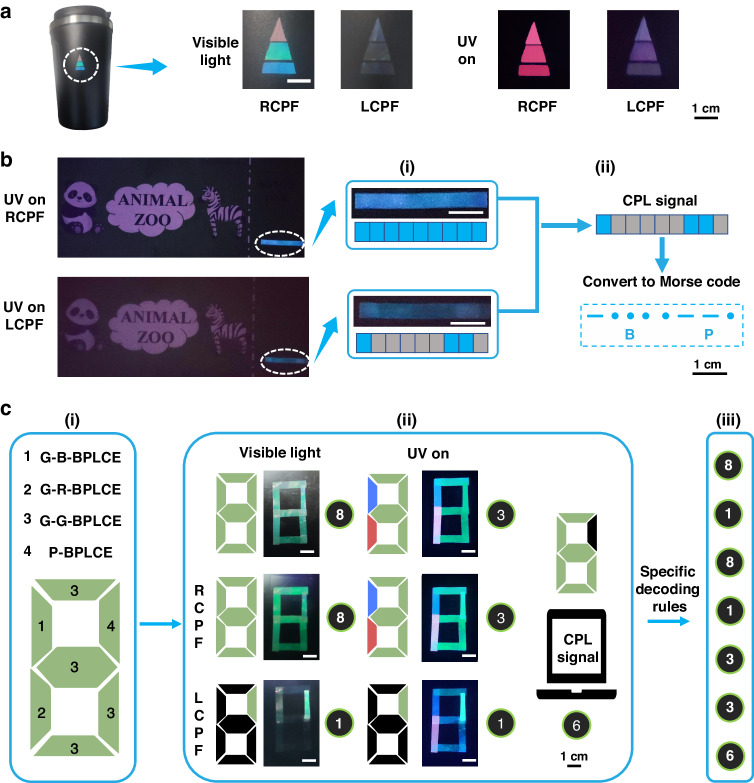
**Anti-counterfeiting and information encryption based on QD-BPLCE. a** Multi-level anti-counterfeiting pattern displayed at different lighting conditions. **b** Single QD-BPLCE film for information encryption and decryption. (i) Photographs of QD-BPLCE under 365 nm light with RCPF and LCPF. (ii) Converting the presence and length of CPL signals into Morse code and identifying information: “BP”. **c** Multiple QD-BPLCE film for information encryption and decryption. (i) Design of the combinational encryption films using G-G-BPLCE, G-R-BPLCE, G-B-BPLCE and P-BPLCE. (ii) Photographs and schematics diagrams of combinational encryption films in various optical states, with the extracted information. (iii) The final information detected under specific decoding rules

The fantastic optical and mechanical characteristics of the QD-BPLCE enable multiple potential applications. In the first case, a designed pattern was applied to a thermos for authentication (Fig. 6a). The pattern features four-level anti-counterfeit measures that are activated by distinct optical states, easily detectable to the naked eye. The first level can be observed under visible light with RCPF, revealing a red, green, and blue triangle. When LCPF is applied, the selective reflection of QD-BPLCE renders the triangle transparent, showing only the color of the bottle’s bottom. The third level is displayed under 365 nm light with an RCPF, exhibiting red CPL thanks to the QDs. In the presence of LCPF and under 365 nm light, the fourth level becomes observable, facilitating the easy differentiation of the polarization states of the emitted CPL intensity. Such an anti-counterfeiting pattern supports four optical states, making counterfeiting difficult and practically impossible. Next, the application of a single QD-BPLCE in the information storage field was investigated. Information was written on partially stretched QD-BPLCE and then fixed by holding at 110 °C for 2 h (Fig. S22). As shown in Fig. 6b, QD-BPLCE with CPL signal disappearance in a specific area was obtained after partial stretching and thermal programming. By detecting the presence or absence of CPL signals and their lengths, they corresponded to Morse code, and the information “BP” was read out. Ultimately, we have also achieved multi-level information encryption by utilizing the differences in optical properties of QD-BPLCE. As shown in Fig. 6c, we combined four types of QD -BPLCE by thermal welding. They all reflect green under visible light and RCPF, indicating the readout information is 8. Under natural LCPF, only thermally treated P-BPLCE reflects green, indicating that the readout information is 1. Without CPF or with RCPF, the strong green CPL forms the number 3 under 365 nm light irradiation. Only P-BPLCE has strong green CPL under LCPF with 365 nm light irradiation, indicating that the readout information is 1. Only P-BPLCE shows no CPL signal, indicating that the readout information is 6. By obtaining the specific arrangement code sequence, we can correctly read out the information set as 8383116.

## Discussion

In conclusion, we have developed solid-state CPL-active materials based on QDs-doping BPLCEs. Unlike the CLC-based CPL materials, the strong CPL generation with high *g*_lum_ values in QD-BPLCE originates from the 3D cubic superstructure and chirality environment independent of PBG modulation. Visualized full-color CPL with a |*g*_lum_| value of up to 0.74 is achieved by doping BPLCEs with red, green, and blue QDs emitters. Furthermore, the 3D chiral structure of QD-BPLCE can be disrupted under mechanical force, enabling dynamic modulation of CPL signals from the on to the off state. Additionally, based on the thermal programming and welding function of dynamic disulfide bonds of the BPLCEs, as well as the distinct CPL characteristic in QD-BPLCE, multi-dimensional anti-counterfeit, information storage, and encryption can be achieved. This study demonstrates the potential of developing CPL functional materials through photonic structures of BPLCEs, suggesting the advancement of BPLCEs-based CPL-active materials for optical coding and information storage applications.

## Materials and methods

### Preparation of QD-BPLCE films

First, the LC precursors were dissolved in dichloromethane (DCM) to form a uniform solution. Then, LC precursors were obtained after evaporation of the solvent in a vacuum oven for 10 h at 35 °C. LC precursors were heated to an isotropic state (45 °C) and filled into the LC cells by capillary forces in the heating stage. To generate large BP platelets, all the samples were slowly cooled at a rate of 0.02 °C min^−1^ until BPI was formed. Then, to achieve thermodynamic equilibrium at the BPI stage, the temperature was maintained at a specific temperature for 1 h. The sample was then exposed to UV light (*λ* = 365 nm) at 30 mW cm^−2^ for 5 min to ensure complete polymerization. After polymerization, the LC cells were opened, and the QD-BPLCE films were obtained.

### Preparation of CLCE films

Following an approach similar to the preparation of QD-BPLCE films, the LC precursors were cooled to the cholesteric phase and then exposed to UV light at an intensity of 30 Mw cm^−2^ for 5 min to ensure complete polymerization. After polymerization, the LC cells were opened, and the CLCE films were obtained.

### Characterization

The phase behaviors and micrograph textures were obtained on a polarizing optical microscope (POM, Leica, DM2500P) with a hot stage calibrated at a temperature accuracy of ±0.1 °C (Linkam, THMS-600). Kossel diagrams were captured on POM (LV100NPOL, Nikon) with a Bertrand lens under monochromatic light (400 nm). Reflection spectra were measured on an Avantes AvaSpec-2048 spectrophotometer in the dark at room temperature. Fluorescence spectra were measured on Hitachi F-4500. CPL spectra were measured on JASCO CPL-200. Differential Scanning Calorimetry (DSC) was measured on a Q2000 (TA Instruments, USA) under nitrogen. Stress relaxation experiments with constant strain (*γ* = 1%) at different temperatures were performed on the DHR-2 rheometer. The mechanical properties of the QD-BPLCE films were measured on the universal material testing machine (E44.104. MTS). The scanning electron microscope (SEM) images of the QD-BPLCE films were obtained on Hitachi S-4700. The transmission electron microscope (TEM) images of the QD-BPLCE films were observed on Hitachi HT 7800 with an operated voltage of 100 kV. The QD-BPLCE films were embedded in epoxy resin, cut along (110) of BPI, and then sliced into 50 nm thin sections using an ultramicrotome (LEICA, EM UC7) before being transferred to copper grids.

### Supplementary information

Supplementary Information
