# Supplementary material for: When quantum dots meet blue phase liquid crystal elastomers: visualized full-color and mechanically-switchable circularly polarized luminescence
Source: Light Sci Appl. 2024 Jun 14;13:140. doi: 10.1038/s41377-024-01479-1 (PMC11178798; doi:10.1038/s41377-024-01479-1)
Supplement: Supplementary file 1 — Supplementary Information [file 41377_2024_1479_MOESM1_ESM.pdf]

Supplementary Information

When Quantum Dots Meet Blue Phase Liquid Crystal Elastomers: Visualized Full-Color and Mechanically-Switchable Circularly Polarized Luminescence

Shan Li, [a,†] *Yuqi Tang,* [b,†] *Qingyan Fan*[a], *Ziyuan Li*[a], *Xinfang Zhang*[c], *Jingxia Wang,*[d] *Jinbao Guo,*[a,*] *and Quan Li*[b,c*]

[a]*Key Laboratory of Carbon Fibers and Functional Polymers, Ministry of Education, and College of Materials Science and Engineering, Beijing University of Chemical Technology, Beijing 100029, China.* *E-mail: guojb@mail.buct.edu.cn

[b]*Institute of Advanced Materials and School of Chemistry and Chemical Engineering, Southeast University, Nanjing 211189, China*. *Email: quanli3273@gmail.com

[c]*Materials Science Graduate Program, Kent State University, Kent, Ohio 44242, USA.*

[d]*CAS Key Laboratory of Bio-Inspired Materials and Interfacial Sciences, Technical Institute of Physics and Chemistry, Chinese Academy of Sciences, Beijing 100190, China*

†These authors contributed equally to this work

1. Materials for the fabrication of the QD-BPLCE.

All the solvents and chemicals were obtained from commercial sources and used without further purification. LC monomers of 1,4-bis-[4-(6-acryloyloxyhexyloxy)benzoyloxy]-2-ethylbenzene (RM82) and benzoesure, 4-[[6-[(1-oxo-2-propenyl)oxy]hexyl]oxy]-4-methoxyphenylester (RM105) were purchased from Nanjing Yushan Chemical Co., Ltd. A right-handed chiral dopant (3R,3aS,6aS)-hexahydrofuro[3,2-b] furan-3,6-diyl bis(4-(4-((4-(acryloyloxy)butoxy)carbonyloxy) benzoyloxy) benzoate) (LC756) was purchased from Nanjing Yushan Chemical Co., Ltd. Non-mesogenic crosslinker 1,4-benzenedimethanethiol (BDMT) was purchased from Shanghai Aladdin Biochemical Technology Co., Ltd. Disulfide crosslinker disulfanediylbis(ethane-2,1-diyl) diacrylate (DSDA) was synthesized in the lab. detailed description of the preparation procedure and characterizations was provided in Supporting Information. Three monochromatic CdSe/ZnS QDs were purchased from Wuhan Jiayuan Quantum dots Co., Ltd. (5 mg/mL each in hexane suspension). Photoinitiator Irgacure 651 was purchased from BASF Company.

The chemical structures of the materials for the fabrication of the QD-BPLCE film are shown in Figure S1.

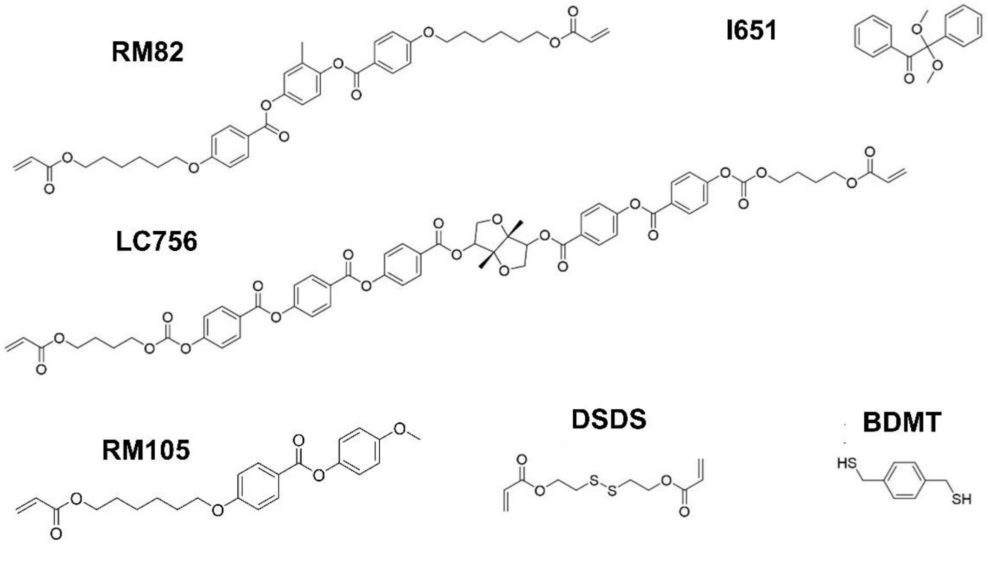

Figure S1. Chemical structures of the components for the fabrication of the QD-BPLCE film.

Synthesis of disulfanediylbis(ethane-2,1-diyl) diacrylate (DSDA):

The synthetic route of crosslinker DSDA is shown in Scheme S1. Bis(2-hydroxyethyl) disulfide (250 mg, 1.62 mmol), TEA (330 mg, 3.24 mmol), and dry THF (4.0 mL) were added into a 25 mL Schlenk flask. Acryloyl chloride (590 mg, 6.40 mmol) was dropwisely added to the solution under a nitrogen atmosphere. The flask was sealed, and the reaction mixture was stirred at 0 °C for 24 h. The mixture was allowed to be warmed to room temperature. The resulting precipitated salt was removed through filtration, and the filtrate was washed with an aqueous sodium carbonate solution (0.1 M). The organic layer was dried over $MgSO_4$ and concentrated on a rotary evaporator. The crude oil was purified by flash column chromatography (petroleum ether: ethyl acetate = 5/1) for the desired product (400 mg, 97 %). ^{1}H NMR spectra were recorded on a Bruker AVANCE III (400 MHz 1H) spectrometer, and the spectra were illustrated in Figure S2. ^{1}H NMR (400 MHz, $CDCl_3$) δ 6.46 (dd, J = 17.3, 1.4 Hz, 2H), 6.16 (dd, J = 17.3, 10.4 Hz, 2H), 5.88 (dd, J = 10.4, 1.4 Hz, 2H), 4.45 (t, J = 6.6 Hz, 4H), 3.00 (t, J = 6.6 Hz, 4H).

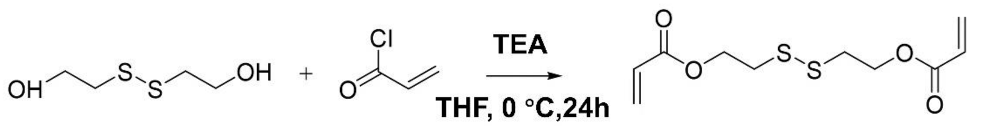

Scheme S1. The synthetic route of crosslinker DSDA.

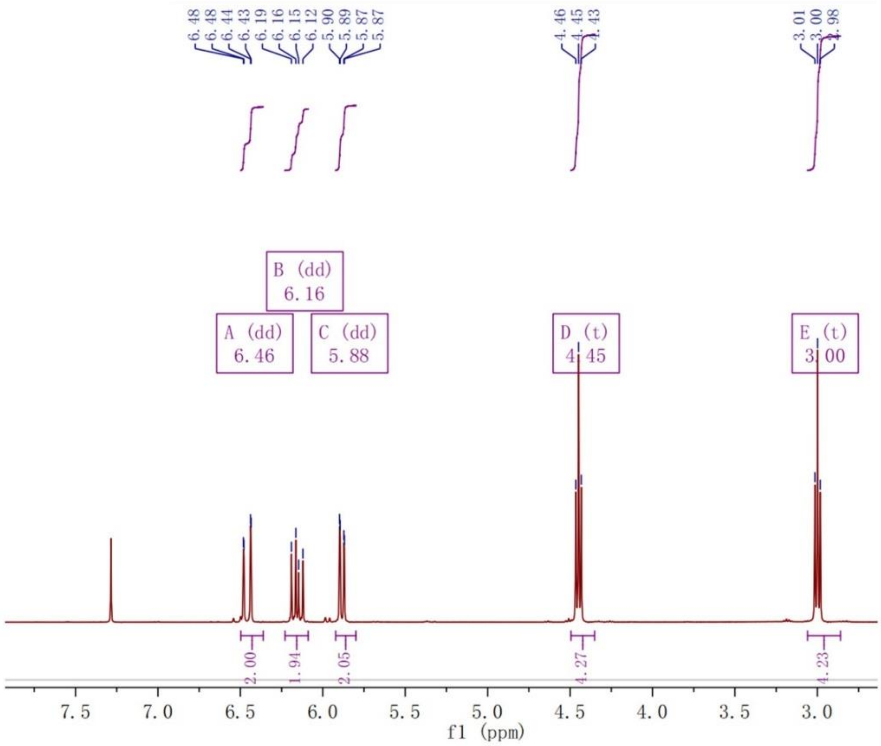

Figure S2. The ^{1}H-NMR spectrum of crosslinker DSDA.

Table S1. Chemical compositions (wt%) of BP pre-mixtures in five different mixtures.

Samples / Materials	R-G-BPLCE	G-G-BPLCE	B-G-BPLCE	G-R-BPLCE	G-B-BPLCE
RM82	55.54	55.0	54.46	55.0	55.0
RM105	23.86	23.5	23.14	23.5	23.5
LC756	4.8	5.7	6.6	5.7	5.7
DSDA	6.4	6.4	6.4	6.4	6.4
BDMT	7.9	7.9	7.9	7.9	7.9
I651	1.0	1.0	1.0	1.0	1.0
QD	0.2(G)	0.2(G)	0.2(G)	0.2(R)	0.2(B)

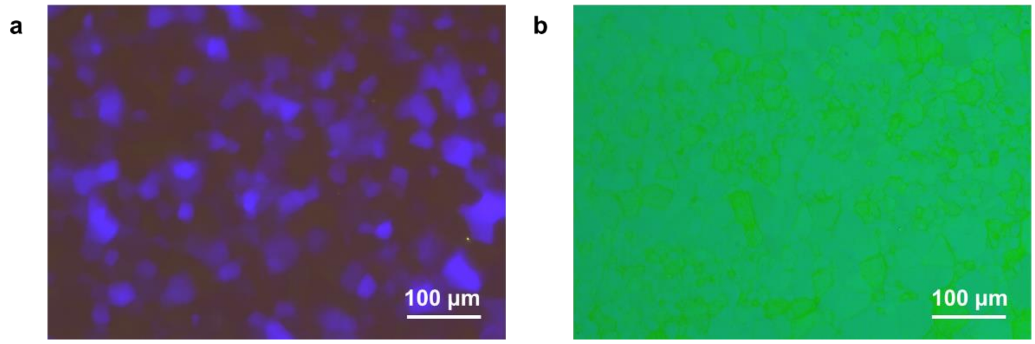

Figure S3. POM images of precursor in the cooling process (a) 33.0 °C-BPII (b) 27.0 °C-BPI.

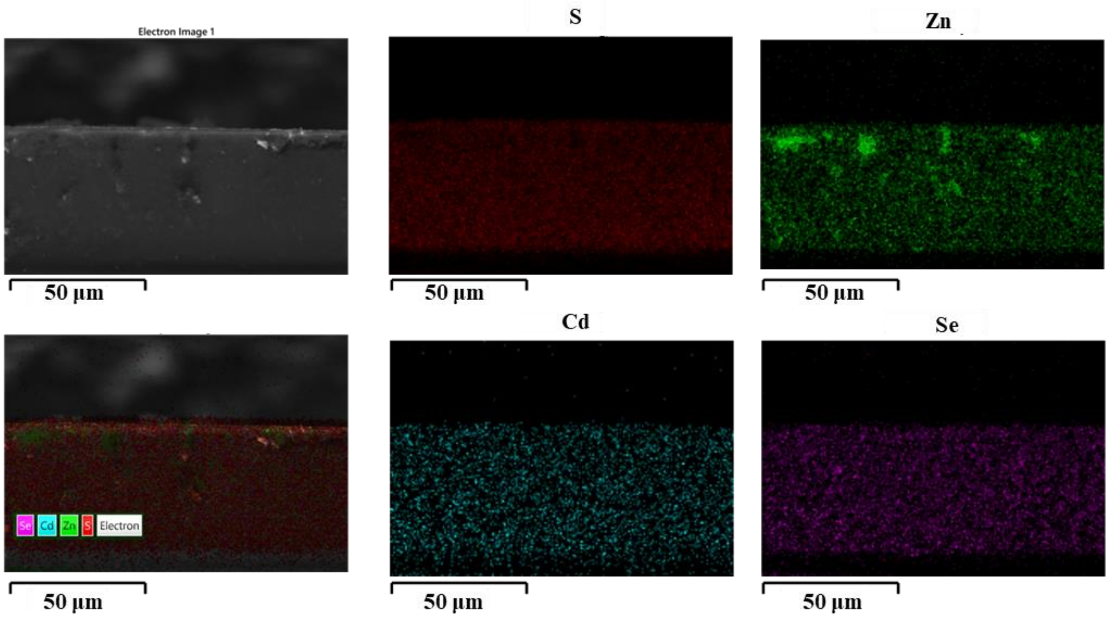

Figure S4. SEM elemental maps of G-G-BPLCE.

2. Thermal stability of QD-BPLCE

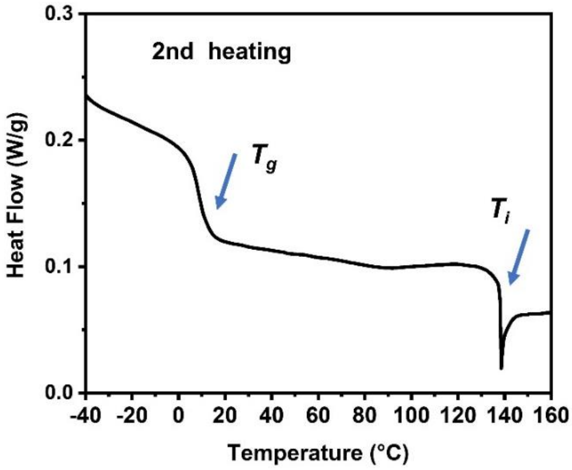

Figure S5. DSC curves of the G-G-BPLCE film at a rate of 10 °C /min under a nitrogen atmosphere during the second heating cycle.

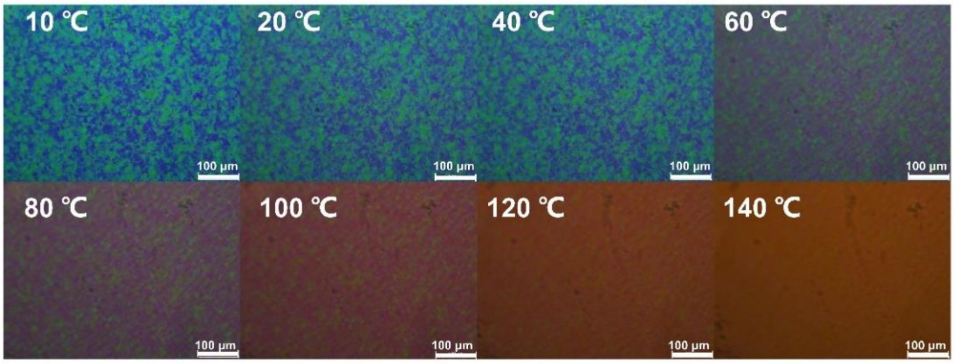

Figure S6. POM images of G-G-BPLCE during the heating process at different temperatures.

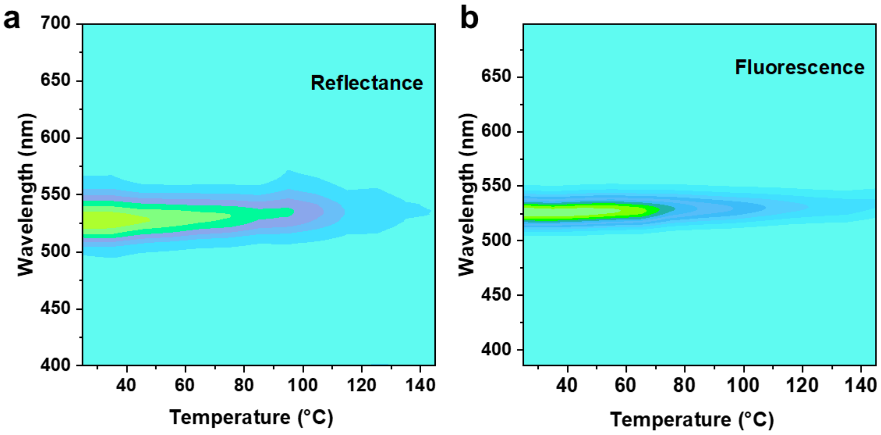

Figure S7. (a) The reflection and (b) fluorescence spectra of G-G-BPLCE from 20 °C to 140 °C.

3. Factors affecting the CPL signal of QD-BPLCE

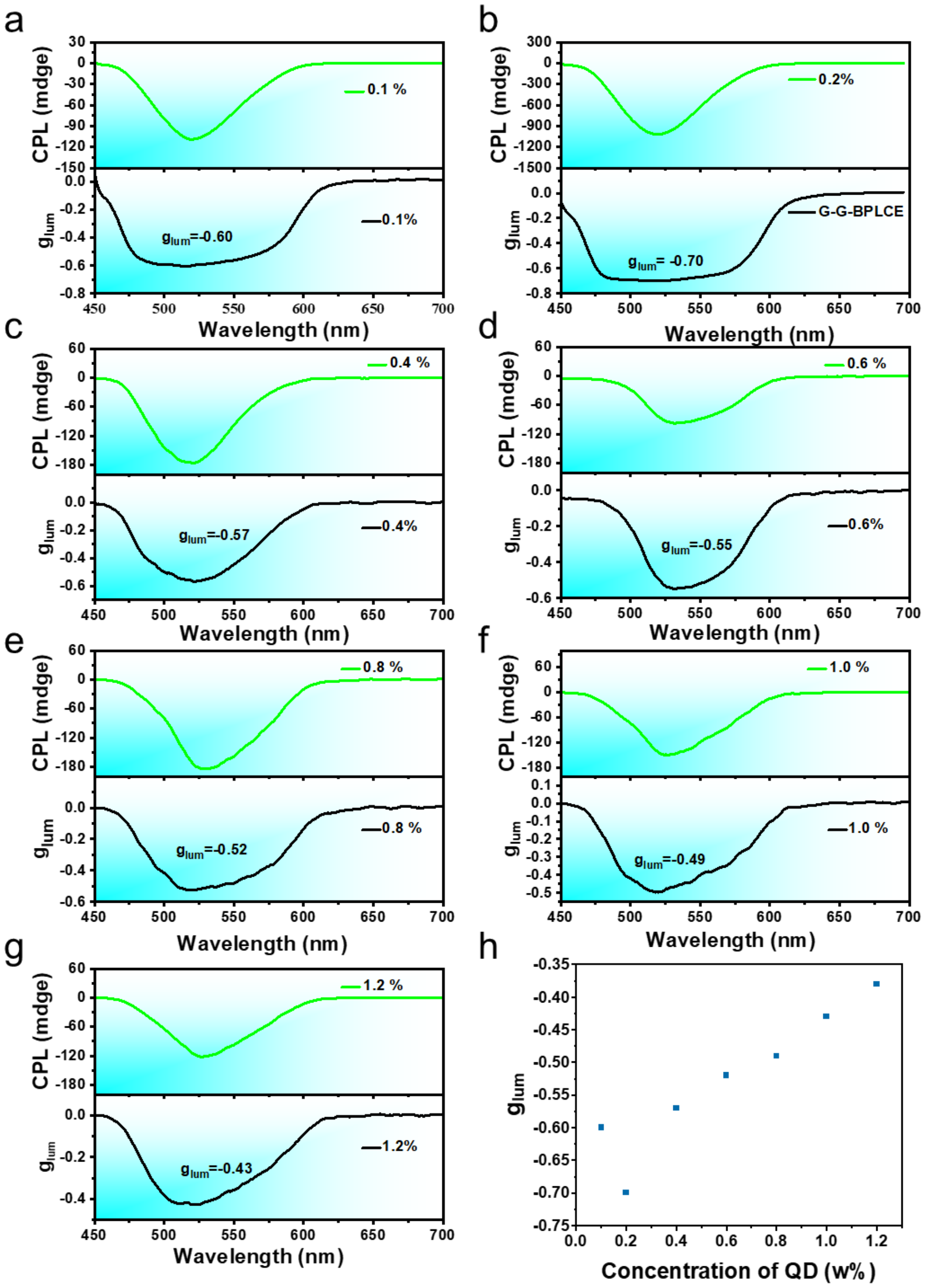

Figure S8. CPL spectrum of materials with QD contents of (a) 0.1 wt%, (b) 0.2 wt%, (c) 0.4 wt%, (d) 0.6 wt%, (e) 0.8 wt%, (f) 1.0 wt%, and (g) 1.2 wt%, respectively. (h) Concentration of QD-dependent g_{lum} of G-G-BPLCE.

When the sample was too thin, the uniform distribution of QDs within the BPLCEs was hindered, resulting in a weaker CPL signal. On the other hand, when the film was too thick, it would compromise the alignment and orientation of the materials, leading to a

decrease in the CPL signal.

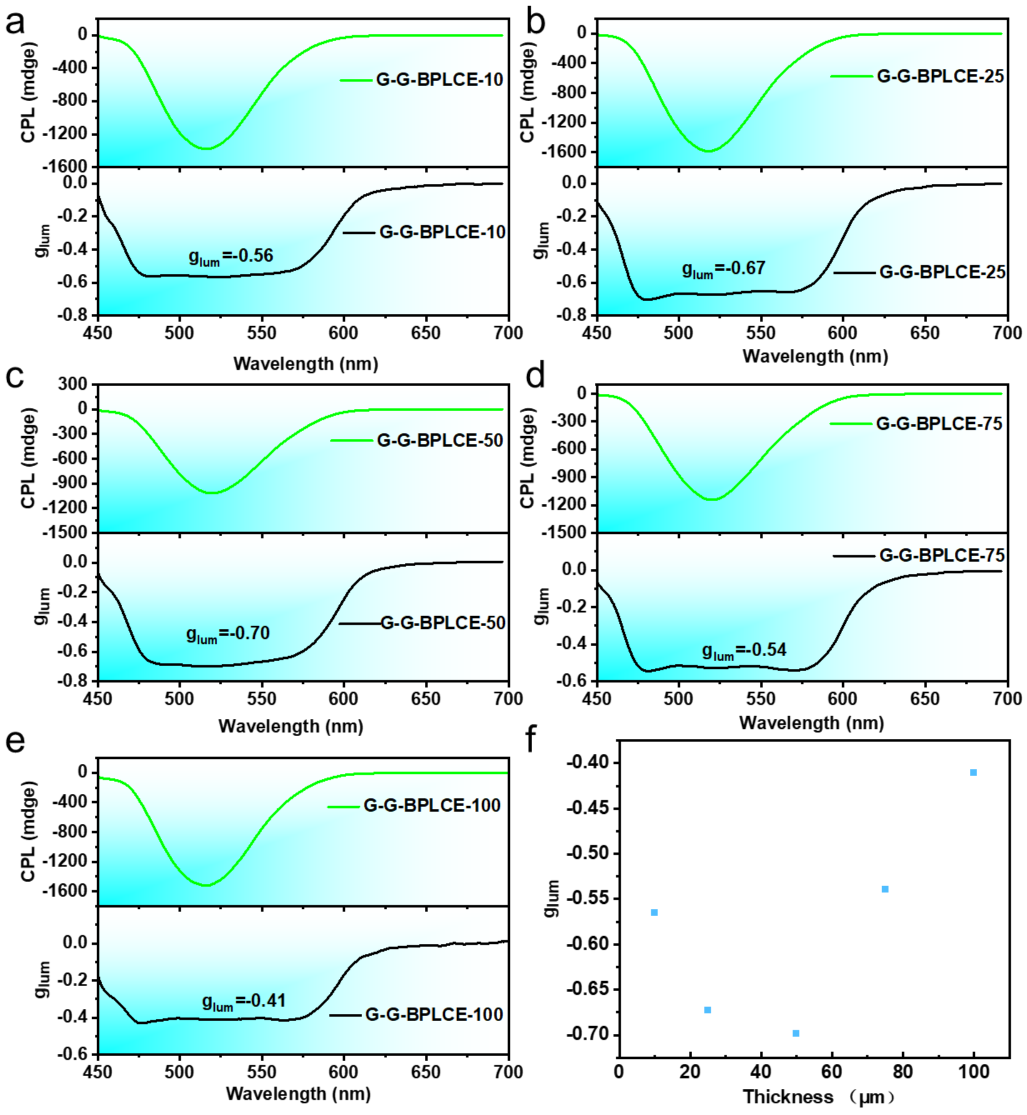

Figure S9. CPL spectrum of G-G-BPLCE with different thicknesses: (a) 10 μm, (b) 25 μm, (c) 50 μm, (d) 75 μm and (e) 100 μm. (f) thickness-dependent g_{lum} of G-G-BPLCE.

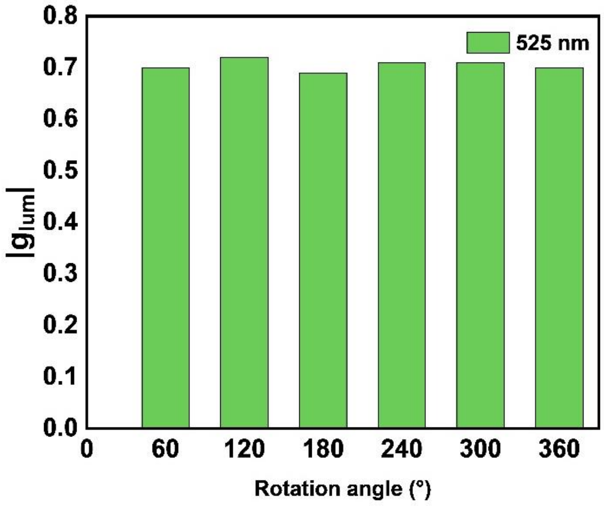

Figure S10. The g_{lum} of G-G-BPLCE by changing the angle of the sample along the direction of incident light propagation.

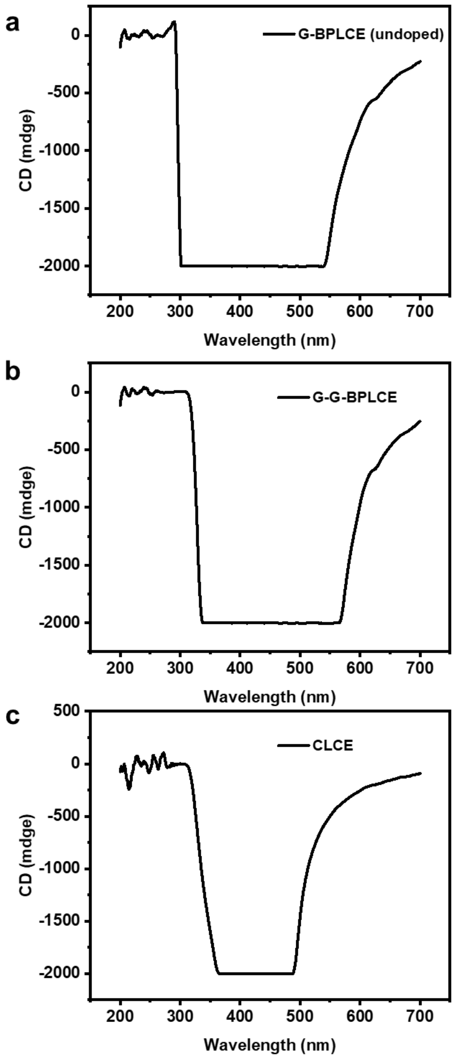

Figure S11. CD spectra of (a) G-BPLCE (undoped), (b) G-G-BPLCE, and (c) CLCE.

4. Mechanical properties of QD-BPLCE

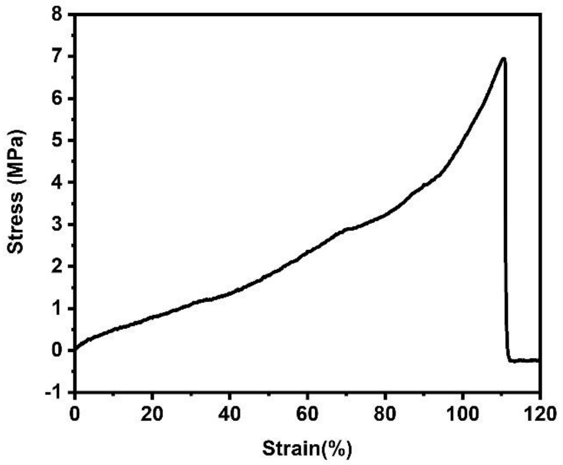

Figure S12. Stress-strain curves of R-G-BPLCE.

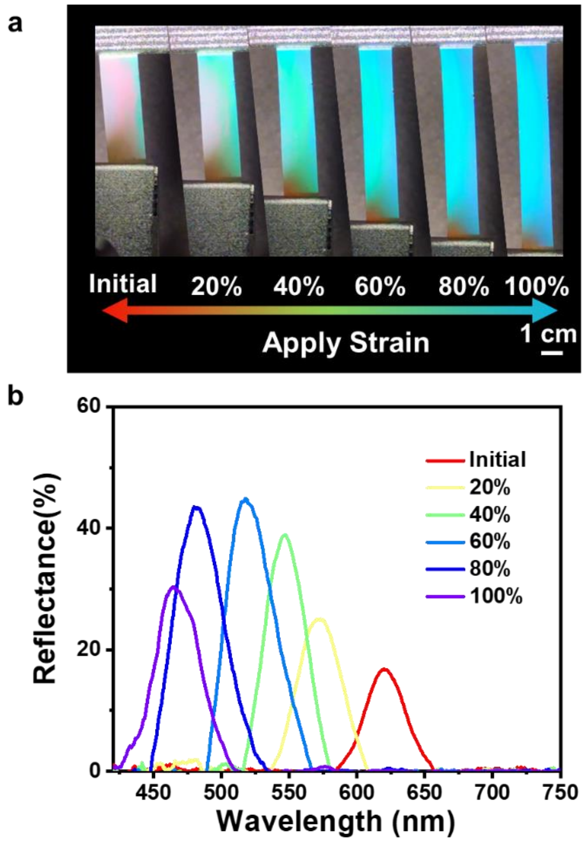

Figure S13. (a) Photographs of R-G-BPLCE being mechanically stretched. (b) Reflection spectra of the R-G-BPLCE at various strains (from 0% to 100%).

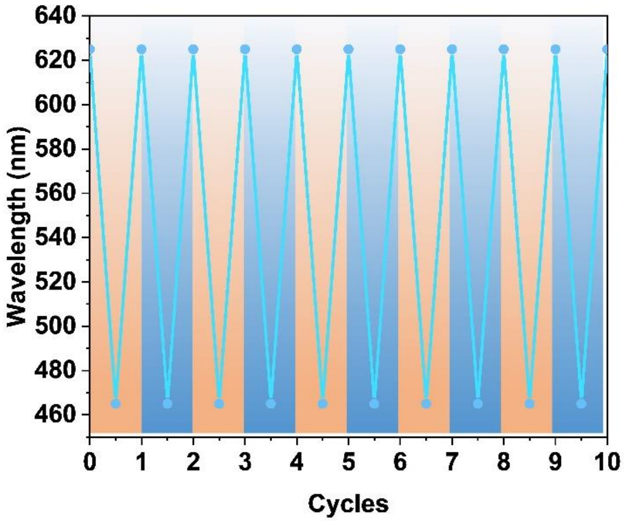

Figure S14. The test for the reversibility of the R-G-BPLCE under 100% deformation for 10 cycles.

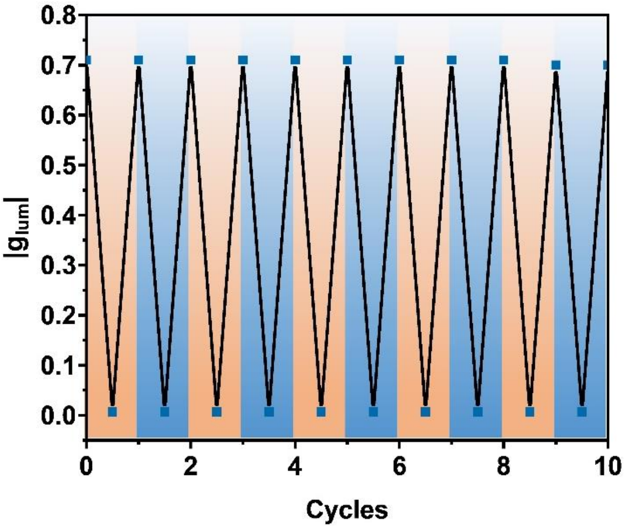

Figure S15. The cyclic performance of stretch-induced disappearance of CPL signals.

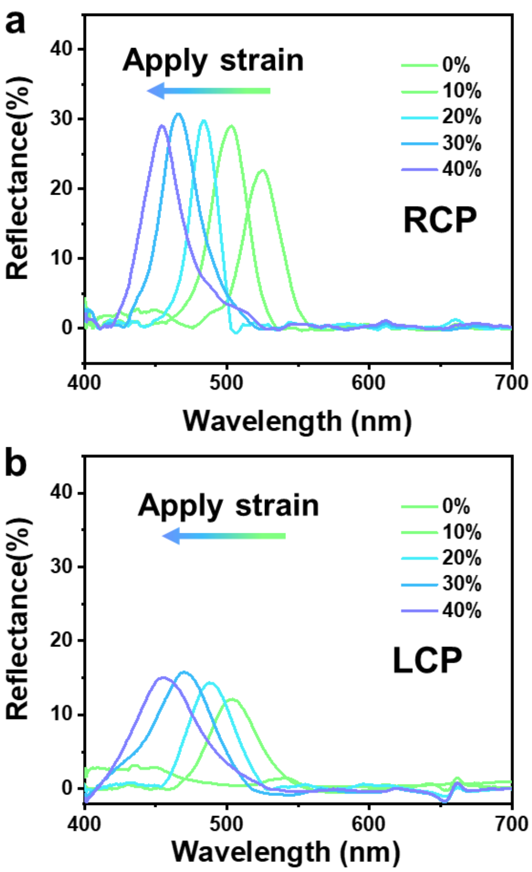

Figure S16. Reflection spectra of the G-G-BPLCE film at various strains (from 0% to 40%) with right-handed circularly polarized filter (RCPF)(a) and left-handed circularly polarized filter (LCPF)(b), respectively.

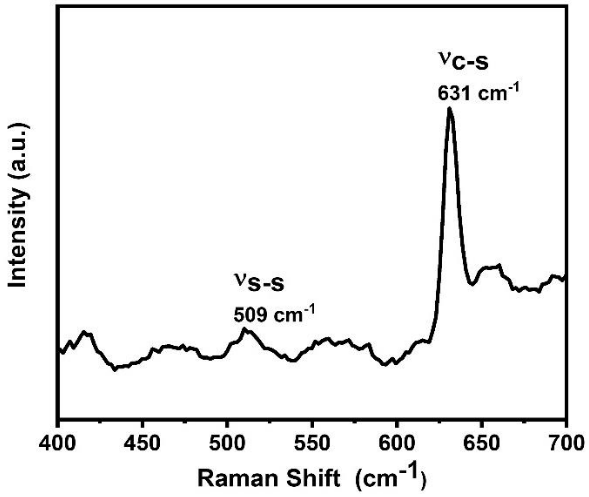

Figure S17. Raman spectra of QD-BPLCE.

5. Calculation of activation energies (E_a)

Stress relaxation experiments were performed on the DHR-2 rheometer with a constant strain (γ=1%) at varying temperatures. As shown in Figure 4c, the relaxation time τ* of QD-BPLCE films was determined at 110 °C to 130 °C. The relaxation time is plotted against 1000/T and fits the Arrhenius relationship in Equation S1.

$$\tau^*(T) = \tau_0 e^{Ea/RT} \quad \text{(Equation S1)}$$

Where τ_0 is the relaxation time at infinite T, Ea is the activation energy of the transesterification (kJ mol^{-1}), R is the universal gas constant (8.314 J K^{-1} mol^{-1}), and T is the temperature (K). Equation S1 can be transformed to Equation S2.

$$ln\,\tau^*(T) = ln\tau_0 + Ea/RT \quad \text{(Equation S2)}$$

According to Figure S10, we obtained Equation S3 and calculated the activation energy to be 90.29 kJ mol^{-1}.

$$ln\,\tau^*(T) = -19.52 + 10.86 * 1000/T \quad \text{(Equation S3)}$$

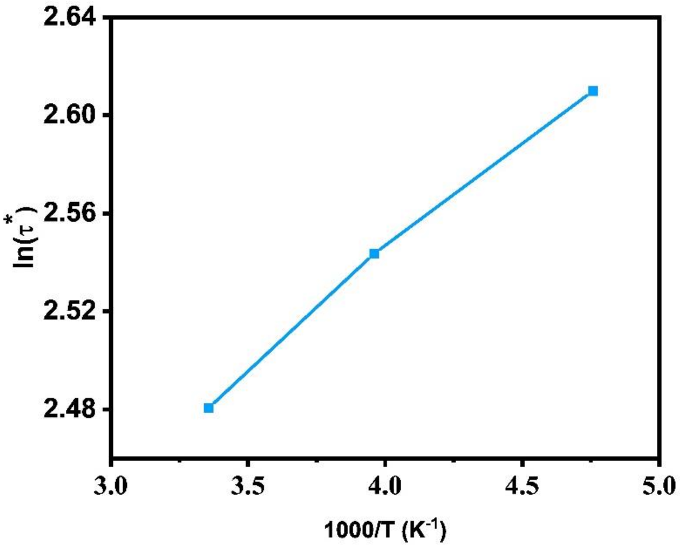

Figure S18. Arrhenius plot of the measured relaxation time for QD-BPLCE films.

6. Welding performance of QD-BPLCE

In the current system, the content of disulfide bonds is relatively low, and to ensure the ordered structure of QD-BPLCE, the processing temperature cannot exceed the phase transition temperature (140 °C), which is lower than the traditional disulfide bond

processing temperature (200 °C). Therefore, the welding of QD-BPLCE must be carried out under the condition of an external catalyst.[1] 1,8-Diazabicyclo [5.4.0] undec-7-ene (DBU) was the catalyst that accelerated the disulfide bond exchange rate. DBU (10 mg, 0.46 mmol) was dissolved in 10 mL dimethyl sulfoxide as the "glue." A few microliters of the above catalyst were lightly brushed onto the interface between overlapping QD-BPLCE using a capillary. The film was welded at 110 °C for one hour to achieve film welding. Figure S20 (Supporting Information) shows the SEM image of the cross-section of the overlapping part before and after film welding. Before welding, there were significant gaps between the films, indicating that the two films were independent. After welding, the two layers were connected into a complete unit, meaning the two films were successfully welded together. Figure S21 (Supporting Information) shows the mechanical properties of QD-BPLCE before and after welding. Compared with the unwelded film, the elongation at the break of the welded film they decreased to 80%. The mechanical performance weakening may be caused by QD-BPLCE being welded in a parallel overlapping way, which also needs to consider the influence of shear modulus in the stretching process.

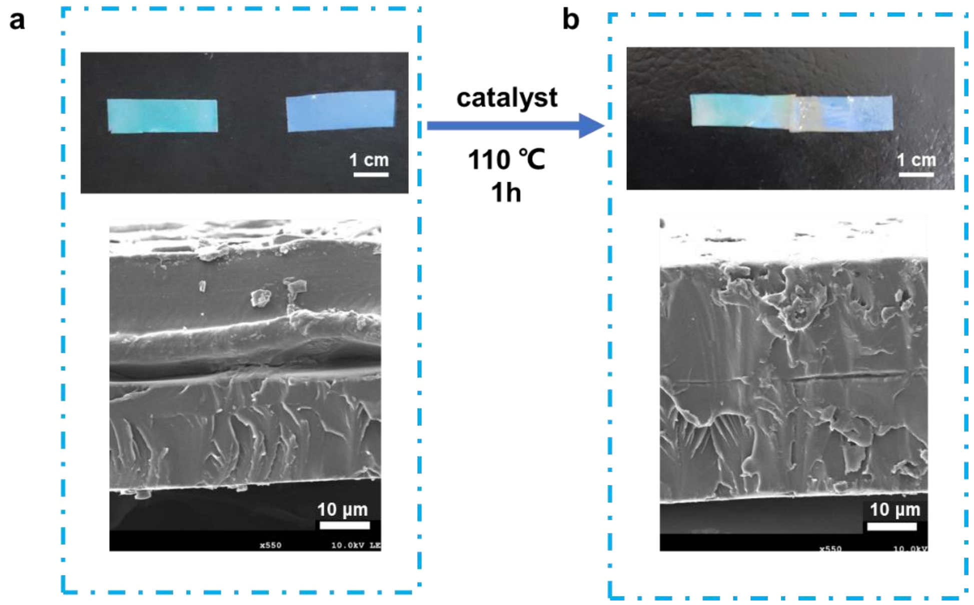

Figure S19. Photographs and SEM images of the cross-section (a) before welding and (b) after welding.

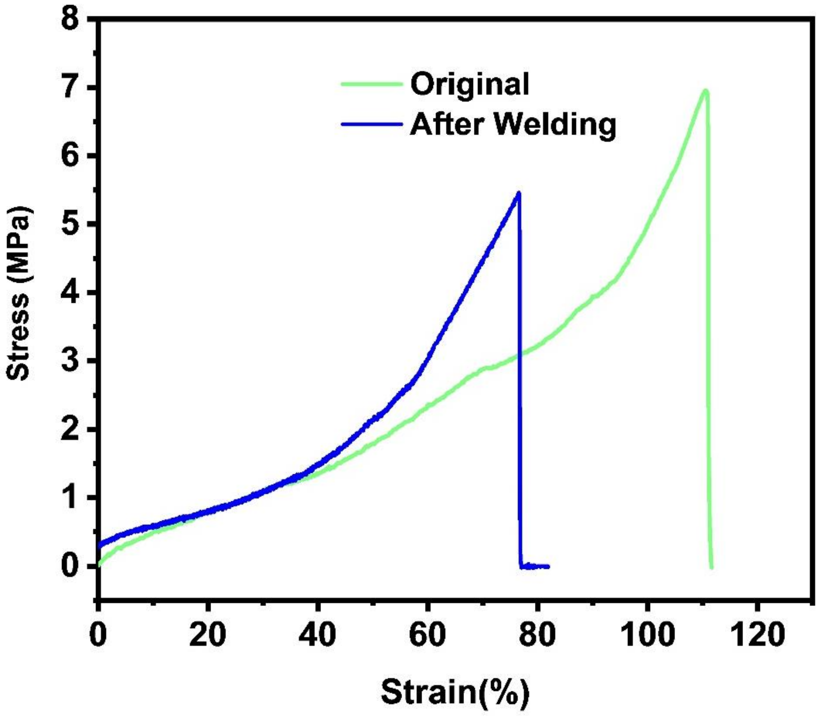

Figure S20. The stress-strain curve of QD-BPLCE in its original state and after welding.

7. The CPL signal of the film after heat treatment without stretching

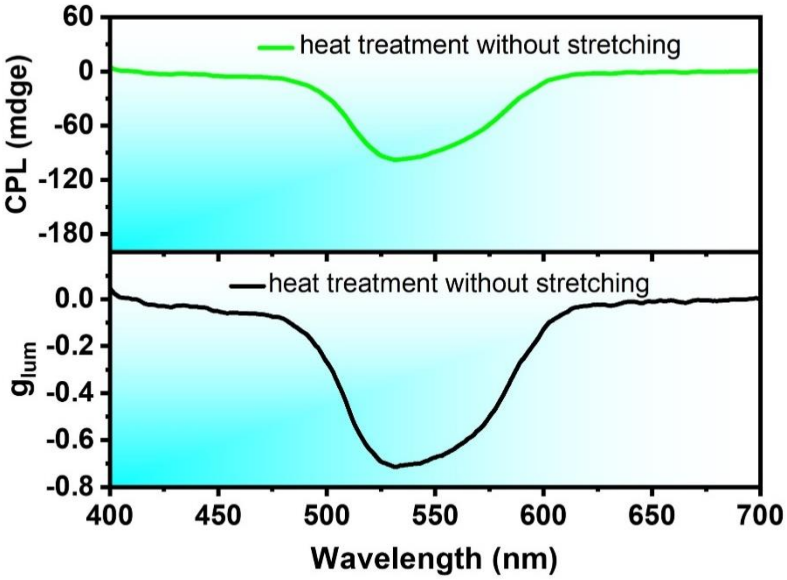

Figure S21. CPL spectra and glum curves of G-G-BPLCE after heat treatment without stretching.

8. Preparation of partially stretched QD-BPLCE

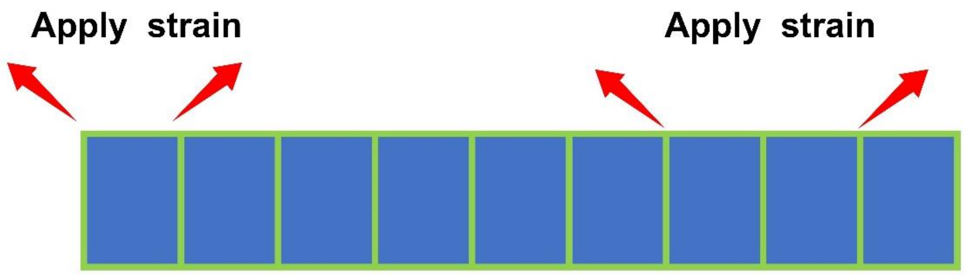

Figure S22. Schematic diagram of the preparation of partially stretched QD-BPLCE.

References:

(1) Chen, L. et al. A cut-and-paste strategy towards liquid crystal elastomers with complex shape morphing. *Journal of Materials Chemistry C* **2018,** *6*, 8251-8257.
